# Optimization of 3D Cooling Channels in Plastic Injection Molds by Taguchi-Integrated Principal Component Analysis (PCA)

**DOI:** 10.3390/polym15051080

**Published:** 2023-02-21

**Authors:** Pham Son Minh, Hung-Son Dang, Nguyen Canh Ha

**Affiliations:** HCMC University of Technology and Education, Ho Chi Minh City 71307, Vietnam

**Keywords:** injection molding, mold temperature control, 3D cooling channel, 3D metal printing, cooling channel optimization

## Abstract

Injection molding has become an increasingly widely used method in the production of plastic parts. The injection process can be separated into five steps: mold closure, filling, packing, cooling, and product ejection. Before the melted plastic is loaded into the mold, the mold needs to be raised to a specified temperature, in order to increase the mold’s filling capacity and improve the resultant product quality. One of the easy methods used to control a mold’s temperature is to provide hot water through a cooling channel in the mold, to raise the temperature. In addition, this channel can be used for cooling the mold with cool fluid. This is simple, effective, and cost efficient, involving uncomplicated products. To improve the heating effectiveness of the hot water, a conformal cooling-channel design is considered in this paper. Through heat-transfer simulation using the CFX module in the Ansys software, an optimal cooling channel was defined according to the simulation result, using the Taguchi method integrated with principal component analysis. The comparison of traditional vs. conformal cooling channels revealed higher temperature rises in the first 100 s in both molds. During heating, conformal cooling produced higher temperatures compared with traditional cooling. Conformal cooling demonstrated better performance, with average temperature peaking at 58.78 °C and a range of 63.4 °C (max) to 54.66 °C (min). Traditional cooling resulted in an average steady-state temperature of 56.63 °C and a range of 61.74 °C (max) to 53.18 °C (min). Finally, the simulation results were verified experimentally.

## 1. Introduction

Injection molding is one of the most widely used processing technologies in the plastics industry [1]. The injection molding process involves several key steps to produce a finished part. Firstly, the raw material, usually plastic pellets, is dried and loaded into a hopper which feeds it into the machine. The mold is then closed by the clamping unit to create a sealed mold cavity. The melted plastic is then injected under high pressure into the mold, where it cools and solidifies. Once the plastic has solidified, the mold is opened and the finished part is ejected. Any excess material, such as flash or gate remnants, is trimmed from the part before it undergoes quality inspection to ensure it meets desired specifications. This process is automated and controlled by the injection-molding machine, with the specific details varying based on the material being molded, the mold design, and the desired final product.

In this molding process, after the mold is closed, melted plastic is filled into the mold cavity and core gaps through the runner system on the mold. For thin-wall injection molding products, mold temperature during this filling process is one of the most critical factors impacting strongly on the filling ability of the melt. With high temperature and uniform distribution on the mold surface, the melt flow moves more easily and fills out the mold. Mold heating can be divided into different types, including surface heating by induction heating [2,3], gas heating [4,5] and volume heating [6,7]. For volume heating, hot water or hot oil can be used. With column heating, the mold temperature can be raised to about 90 °C by hot water flowing inside the cooling channel [8]. The mold needs to be heated to a higher temperature for certain difficult filling cases such as thin-wall products or micro-sized products. The hard filling is more critical for material which has a lower viscosity, such as PC-ABS or PMMA. In this case, another fluid such as hot oil can be used to raise the mold temperature. Many methods have been researched and developed in addition to the use of hot water and hot oil to improve the efficiency of mold heating, including electromagnetic induction heating, developed by Chen et al. [9,10], and infrared heating, invented by Hwang et al., which was effective when the mold surface temperature was raised from 80 °C to 188 °C within only 15 s [11]. High-frequency proximity heating was also used in research by Yao, Thomas, and Byung Kim [12]; the results were even more impressive, with the mold cavity being rapidly heated from room temperature to nearly 240 °C within only 5 s [12]. Although all of the above research reported remarkable results in the mold-heating process, considering manufacturing costs, the use of liquid (e.g., hot water, oil) through a cooling channel is still considered the mainstream solution. The issue that needs to be considered in the design of the cooling channel is that the mold must be heated to a high temperature with uniform distribution over its surface. Using a conformal cooling channel instead of a conventional cooling change could provide an effective solution to this problem. There have been many studies on a conformal cooling channels in recent years, focusing on optimizing and evaluating the effectiveness of conformal channels in the cooling process and how they affect product quality. In 2014, Shaiful et al. [13] analyzed warpage when using a straight cooling channel and conformal cooling channel. The simulation was implemented using Autodesk mold flow and results were analyzed by ANOVA and Taguchi. Through simulated changes in injection temperature such as coolant and melt temperatures, packing pressure and packing time, the study compared the percentage of warpage in the straight and conformal cooling channels. The results showed that with the best parameters for each type of cooling channel, the conformal cooling channel produced less warpage than the straight cooling channel (0.2826 mm versus 0.3005 mm). Vojnová [14] studied the benefits of the conformal cooling channel systems in the molding process. A spiral cooling channel was developed and analyzed and it was reported that the cooling time was reduced by up to 20% and production-cycle time by up to 10%. In 2018, Li et al. studied topology optimization to design conformal cooling channels, aiming to use the BEM method to optimize the cooling channel. The optimization starts with a complex network of channels; during the optimization process, the geometric features of the channel network are modified to achieve a better cooling performance. The cooling system’s optimal geometric and topologic structure can be obtained by deleting invalid channel sections [15]. Park, H. S. & Dang, X. developed an injection mold with a conformal cooling channel. This research aimed to find the solution to the uneven cooling and long cycle time in the molding process of an automotive part. The results showed that the conformal cooling channel had more uniform temperature distribution than the traditional cooling channel, and the cooling time was reduced by more than 30% [16,17].

In general, when a conformal cooling channel is integrated into an injection mold, it can result in several changes to the molding process, including reduced cycle time, improved part quality, increased tool life, increased process repeatability, and increased design flexibility. However, the use of conformal cooling channels requires a more complex mold design and manufacturing process and may result in increased costs, so it is important to evaluate carefully the specific needs of a molding application before making a decision. The primary research in this study focuses on optimum cooling-channel parameters through analysis of heat transfer from the cooling channel to the mold, using the CFX module in the Ansys software. The responses considered in this analysis were the average temperature on the mold surface and the temperature difference on the mold surface. The optimization method used in this study was Taguchi-integrated principal component analysis (PCA).

## 2. Simulation and Experimental Methods

### 2.1. Design Products for Experiments

**Figure 1:**
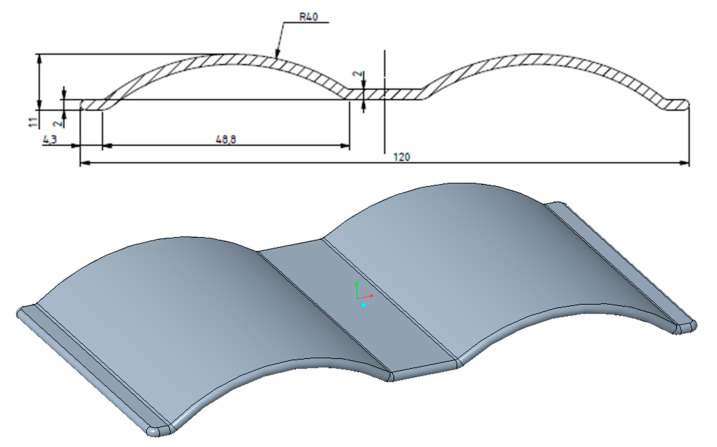
Drawing of experimental product detail.

In order to conduct the most general assessment of the heat transfer capacity of the 3D cooling channel, the part designed for the test must have characteristics that include the ability not to fill out the cavity when injected; 3D profiles should be used for the experimental sample, in order to observe clearly the effect of heat transfer from the conformal cooling channel; and it should not be too complicated with respect to the experimental scale. For the above reasons, the selected experimental product was a flat plate part with a curved profile. The drawing of this product is shown in Figure 1.

### 2.2. Mold Design

**Figure 2:**
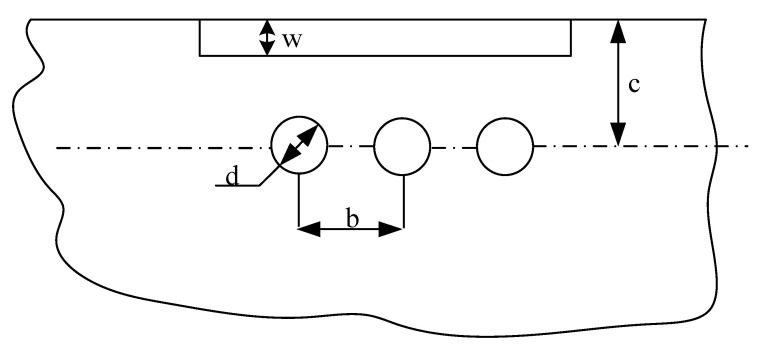
Cooling channel design guidelines.

Mold design was carried out using the Creo parametric software. The purpose of the designed mold was to verify the heat and the effectiveness of the conformal cooling channel, so it was not necessary to consider the number of mold cavities and the number of cavities was selected as one. The conformal cooling channel was designed to make the channel profile closely match the mold profile, maintaining an even distance between the channel and the die surface. The position and dimension of the cooling channel were considered in terms of affecting the thickness of the experimental product. The guidelines for designing a straight cooling channel, shown in Figure 2, can be used as a reference. These parameter details were shown in Table 1. 

**Figure 3:**
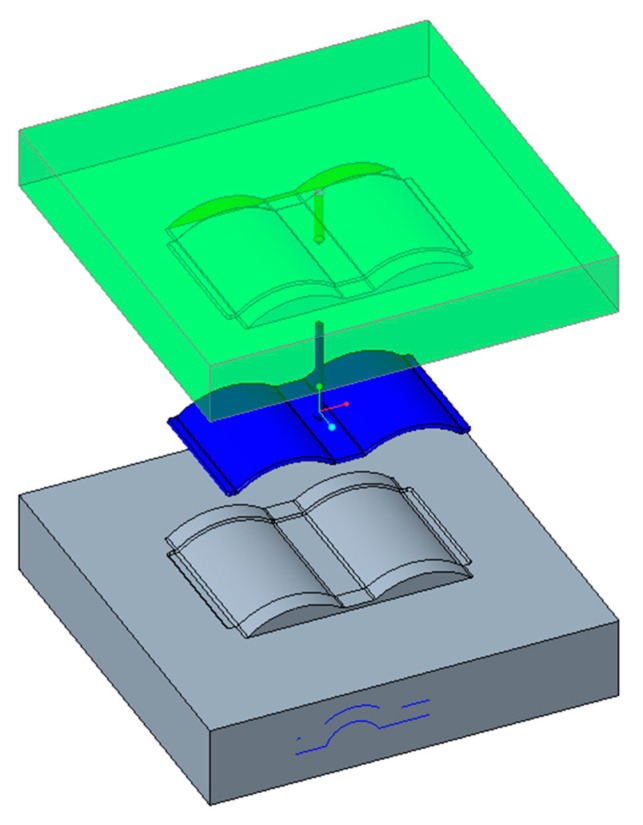
CAD model of injection mold.

**Figure 4:**
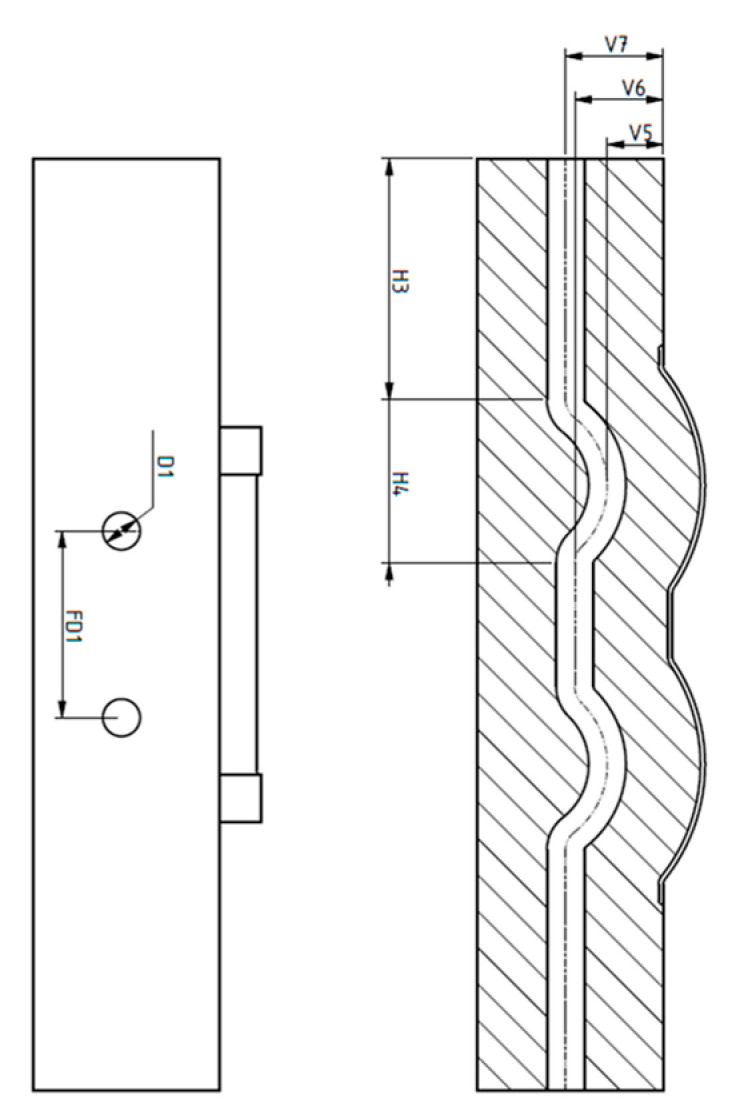
Design parameters for the conformal cooling channel.

A CAD model of a generic injection mold is shown in Figure 3. A proposal for the design parameters of the conformal cooling channel was constructed using the design guidelines for cooling channels. Seven parameters were set to define the full shape of the cooling channel, including channel distance, diameter, length, and distance from the center line of the cooling channel to the mold surface. The design of the cooling channel is shown in Figure 4, and the parameter ranges are defined in Table 2. 

### 2.3. Simulation Setup

**Figure 5:**
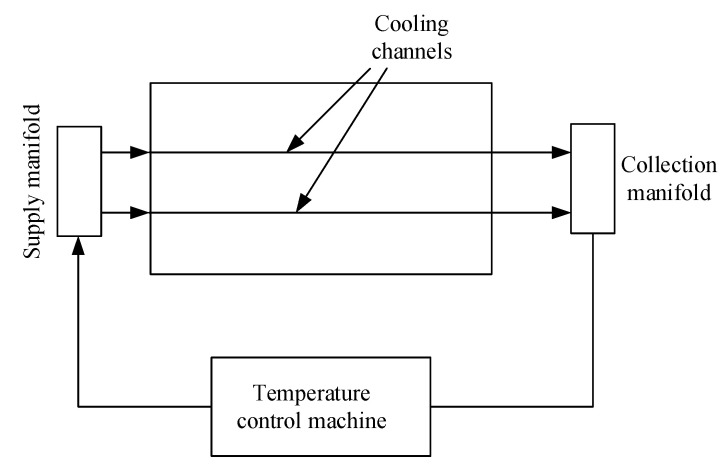
Diagram of components used in the heating process.

In this study, the focus is on optimizing the cooling channel by examining the heat transfer between the mold and coolant. The temperature at the mold surface was used to observe this phenomenon, with the assumption that the melt volume is not taken into consideration when heating and cooling the mold. In the heating process, water at an average temperature (approximately 30 °C) from a tank is heated to 90 °C by a temperature-control machine, thus heating the mold when passing through the conformal cooling channel. At the outlet of the cooling channel, the manifold collects the water and passes it through the temperature-control machine again, and the heating cycle is repeated. All components in the heating process are shown in Figure 5. 

**Figure 6:**
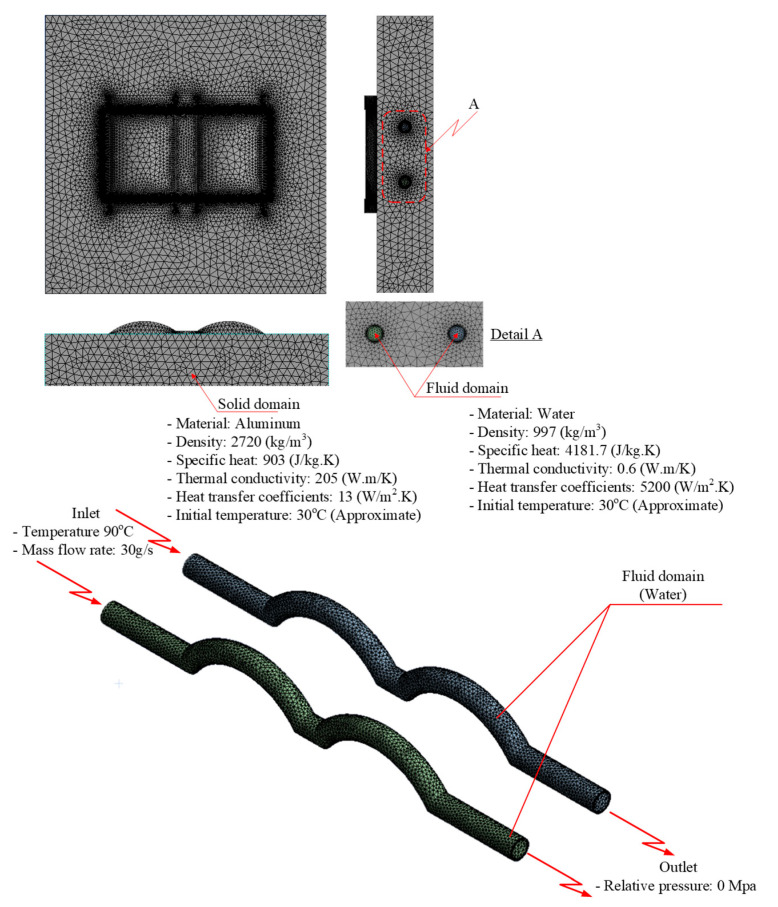
Heat transfer simulation domain.

This process was applied in the simulation. Without loss of generality and simplification, the simulation focused on the cavity. We included two kinds of domain in the simulation: a solid domain (i.e., the mold) and two fluid domains (i.e., two cooling channels separated into two domains). The two fluid domains were used in the same setting. The inlet temperature for the fluid domain was 90 °C. For the solid domain, the material used for the simulation was aluminum. This simulated heat-transfer method included conduction from hot water in the conformal cooling channel to the mold surface and free convection from the mold surface to the ambient region (ambient temperature approximate 30 °C). The thermal properties of each material used in the simulation are given in Table 3 [13], and the heat-transfer scheme is shown in Figure 6. 

**Figure 7:**
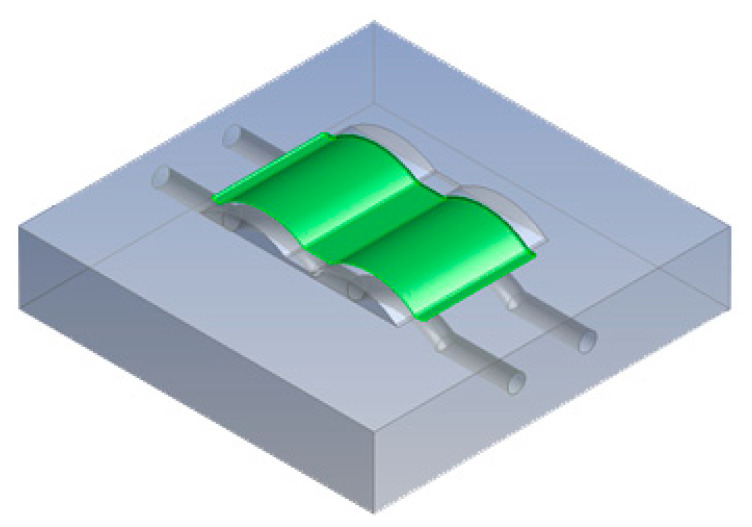
The measurement surfaces.

The aim of this study was to monitor the temperature control of the mold using a conformal cooling channel. To achieve this, the temperature at the completion of a heating or cooling cycle was recorded. The simulation results show the average temperature and the temperature variation between the maximum and minimum temperatures. The temperature was measured at the surface of the mold in contact with the part, as shown by the green surface in Figure 7.

### 2.4. Design of Experiments for Taguchi and Principal Component Analysis (PCA)

**Figure 8:**
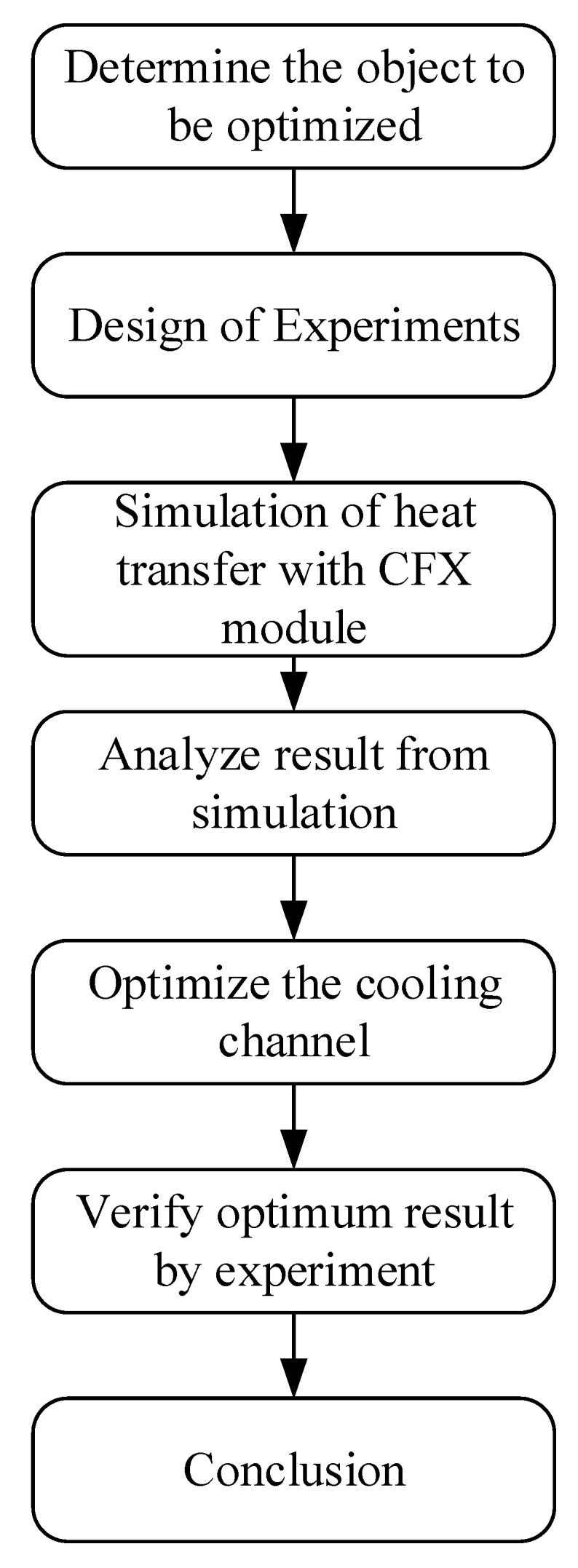
Optimization flow chart.

The flowchart describing this study is shown in Figure 8, below. Design of experiments (DOE) is an experimental strategy designed to determine the relationship between input parameters and responses. Many types of DOE have been developed, such as full factorial design, fractional factorial design, Taguchi orthogonal, Box–Behnken, and so on. The Taguchi method is a separated type of fractional factorial design, which uses an orthogonal array to construct experiments. This orthogonal array is defined based on a factor’s total Degrees of Freedom (DOFs). The DOFs of all factors in this experiment are shown in Table 4.

The total DOFs of all factors was 15 and, so, the number of experiments must be larger than 15. The number of factors being 7 and 3 levels for each factor, the orthogonal array L32 (2^1 4^9) was chosen. The DOE is provided in Table 5.

Taguchi’s method focuses on product quality. In Taguchi’s method, product quality must be designed in parallel with product design, not with inspection after release of the product. The Taguchi method separates product characteristics into three types, and the optimum is determined based on the Signal-to-Noise ratio. The three types of Taguchi Signal-to-Noise ratios include [18,19,20]:

− The bigger, the better:

(1) $$SN=-10\;log\left[\frac{1}{n}\overset{n}{\underset{i=1}{\sum }}\frac{1}{y_{i}^{2}}\right];$$

− The smaller, the better:

(2) $$N=-10\;log\left[\frac{1}{n}\overset{n}{\underset{i=1}{\sum }}y_{i}^{2}\right];$$

− The closer to the nominal valuer, the better:

(3) $$SN=-10\;log\left[\frac{1}{n}\overset{n}{\underset{i=1}{\sum }}{\left(y_{i}-y_{n}\right)}^{2}\right].$$

There are many approaches that can be combined with the Taguchi method to address multi-response problems. Engineering judgment was the earliest method used with the Taguchi method. In particular, Reddy et al. combined the Taguchi method and engineering judgment to optimize injection parameters, considering three responses [21]. However, it seems that the reliability of this method is not high. In 2006, Jeyapaul et al. researched the combination of the Taguchi method with a GA for multi-response optimization. Here, the weight for each response is considered a gene, where the sum of weights is equal to one. Then, the multiple normalized SN ratio values are combined into a single performance measure (WSN) using the optimal weights [22]. This combination has shown excellent results, but the process for this method is very complex. Another proposal for this problem involves the assignment of weight for each response; for example, Shiau assigned a weight to each response, and then combined these weights. For instance, if there are two responses with the S/N ratio S/N_1_ and S/N_2_, and the weights corresponding to each ratio are $w_{1}\;\mathrm{and}\;w_{2},$ respectively, then the combined ratio is $L=S/N_{1}*w_{1}+S/N_{2}*w_{2}$. The value *L* can be used as a total quality loss for optimization [23]. The same idea of weight assignment can be applied using principal component analysis (PCA) in combination with the Taguchi method for multi-response optimization problems. Antony, in 2000, used Taguchi’s loss function and PCA for multi–response optimization in industrial experiments. PCA is generally performed for data exploration, data reduction, and data classification [24]. 

**Figure 9:**
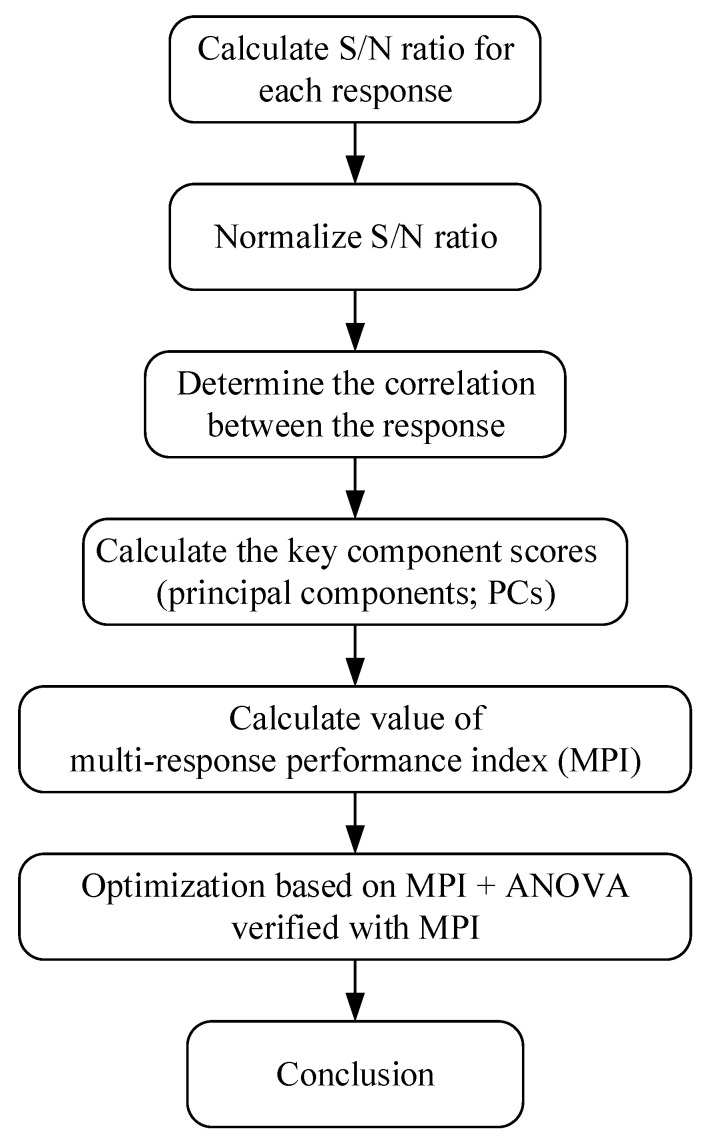
Taguchi-integrated PCA optimization process.

In this method, the S/N ratio is first normalized, following which a weight is assigned for each normalized ratio using PCA. This method is quite simple and easy to implement. Jeyapaul also recommended this approach in his review in 2005 [25]. Grey relational analysis may also be combined with the Taguchi method to solve multi-response optimization problems, as in the research of Haq, A. Noorul, P. Marimuthu, and R. Jeyapaul in 2008 [25]. A grey relational grade is obtained from the grey analysis; then, based on the grey relational grade, optimum levels of parameters are identified, and the significance of contributions of parameters is determined by ANOVA. In this method, the S/N ratio is first normalized, and these normalized values are used to calculate the grey relational coefficient. The grey relational grade is then generated through the grey relational coefficient. Finally, the optimal factors and their combination levels are obtained. A higher grey relational grade implies better product quality [26]. In this research, the Taguchi method integrated with PCA was used for optimization. The process diagram for the use of Taguchi with PCA is shown in Figure 9 [27].

### 2.5. Calculate S/N Ratio for Each Response

The first step of the proposed method is to calculate the S/N ratio for each response, based on Formulas (1)–(3). For average temperature, the characteristic was ‘the more significant, the better’; meanwhile, for temperature difference, the characteristic was ‘the smaller, the better’. The results of the simulation and the S/N ratios are given in Table 6. In the results, P8 denotes the temperature difference, and P9 denotes the average temperature.

### 2.6. Normalize S/N Ratio

The S/N ratio was normalized based on the characteristic. There are two equations for two types of characteristic:

The smaller, the better: [28]

(4) $$X_{i}{\left(k\right)}^{*}=\frac{X_{i}\left(k\right)-minX_{i}\left(k\right)}{maxX\left(k\right)-minX\left(k\right)};$$

The larger, the better: [28]

(5) $$X_{i}{\left(k\right)}^{*}=\frac{maxX_{i}\left(k\right)-X_{i}\left(k\right)}{maxX\left(k\right)-minX\left(k\right)},$$

where

$X_{i}\left(k\right)$ denotes the value of the S/N ratio for response *k* in experiment *i;*

maxX(k) is the maximum value of the S/N ratio for response *k*;

minX(k) is the minimum value of the S/N ratio for response *k*;

$X_{i}{\left(k\right)}^{*}$ is the normalized value of the S/N ratio for response *k*.

### 2.7. Determining the Correlations between the Responses

The Pearson correlation formula was used determine the correlations between the responses. The correlation value measures the relationship between each response, and can be negative, positive, or zero. If the correlation value is negative, the two responses are proportional. If the correlation value is positive, the two responses are inversely proportional. If the correlation value is zero, there is no correlation between the two responses. This value can be easily calculated using statistical software, such as Excel, Minitab, or others. 

The Pearson’s correlation coefficient is calculated as follows:

(6) $$r=\frac{\sum^{\;}\left(x_{i}-\bar{x}\right).(y_{i}-\bar{y})}{\sqrt{\sum^{\;}{\left(x_{i}-\bar{x}\right)}^{2}.\sum^{\;}{\left(y_{i}-\bar{y}\right)}^{2}}},$$

where

$x_{i},y_{i}\;$ are the normalized value of each response in experiment *i*;

$\bar{x},\;\bar{y}\;$ are the means of each response;

*r* is the correlation coefficient.

Using the normalized S/N ratio value, the Pearson correlation coefficient was r = 0.31. This means that the relationship between two responses was proportional: when the average temperature increased, then the temperature difference increased, and vice versa. 

### 2.8. Calculating the Component Scores (Principal Components; PCs)

When conducting PCA, *k* (*k* ≤ *p*) components are obtained, which explain the variance in the *p* responses. Principal components are independent of each other (uncorrelated) [29]. Table 1 shows the explained variation in these two responses and the eigenvalue of each principal component (Table 7). 

The multi-response performance index was calculated based on the formula:

(7) $$MPI=\vartheta_{1}\times 0.546+\vartheta_{2}\times 0.454,$$

where $\vartheta_{1}\;\mathrm{and}\;\vartheta_{2}$ are the principal components corresponding to the responses P8 and P9, respectively.

The principal components for each response and the MPI are given in Table 8.

The mean MPI for each level is given in Table 9.

**Figure 10:**
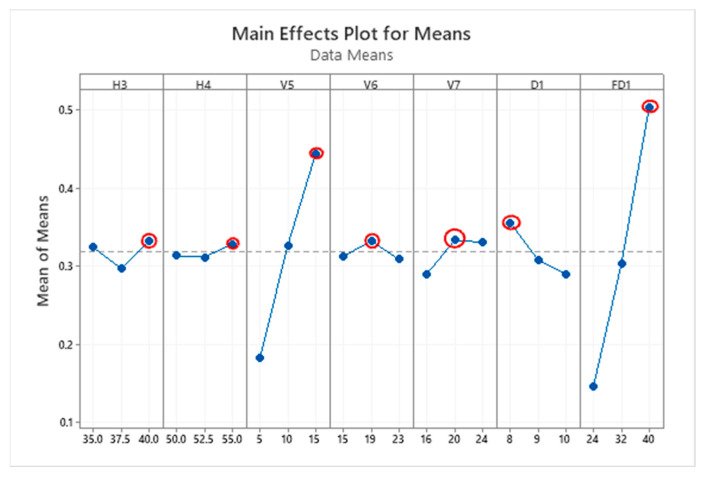
Main effect plot for mean MPI.

The main effect plot for MPI means is shown in Figure 10.

The graph in Figure 10 shows the main effect for mean MPI. The level with the highest mean value is the optimum point and is marked by a red circle on the plot. It is clearly apparent that for the variables H3, H4, V5, and FD1 the optimum value is level 3. Meanwhile, the optimum value of variables V6 and V7 is level 2 and for the remaining variable D1, the optimum point is at level 1. All the optimum levels are listed in Table 10. The optimum value was highlight as red point in Figure 10.

### 2.9. Determining Optimum Parameter Levels

The optimum levels for the parameters, obtained by the MPI mean plot, are given in Table 10.

The process variables with a significant influence and the contribution of the variables to the responses under study were determined through analysis of variance (ANOVA). In this study, ANOVA was performed against the multi-response performance index (MPI) value, representing all responses simultaneously [15]. Table 11 provides the ANOVA results.

In this ANOVA analysis, the significance level (Alpha value—α) is 0.05, meaning that if the *p* value is smaller than α then the differences between some of means are statistically significant, and if the *p* value is larger than α then the differences between some of means are not statistically significant. It can be seen in Table 11 that the variable V5, D1, and FD1 have *p* values smaller than the α value, so these three variables have significant statistical effect. Meanwhile, the *p* values of H3, H4, V6, and V7 are larger than the α indicating that those variables are not statistically significant. Furthermore, the R-square value is 97.15%, which is a high value reflecting the good fit of the model.

The R value of 97.15% indicates that the data were statistically significant. According to the ANOVA table, we can see that the parameters V5, D1, and FD1 presented *p* < 0.05, so these parameters influenced the responses at the 95% confidence level. Furthermore, these levels had F values larger than [F] = 3.37; thus, the DOFs of each factor could obtain the [F] value through the F distribution table, meaning these parameters were adequate for affecting the responses. The remaining factors had F values smaller than [F] and so could be removed from the statistical analysis.

### 2.10. Verifying Optimal Values through Simulation

After obtaining the optimum levels, the simulation was implemented to verify the results. Table 12 and Table 13 show the simulation results. The simulation verified the heating process and demonstrated the cooling effectiveness of the two types of cooling channels. The simulation was implemented using the ANSYS module CFX. The model was created by using the optimum point on the before steps, and boundary conditions were applied as in Figure 6.

**Figure 11:**
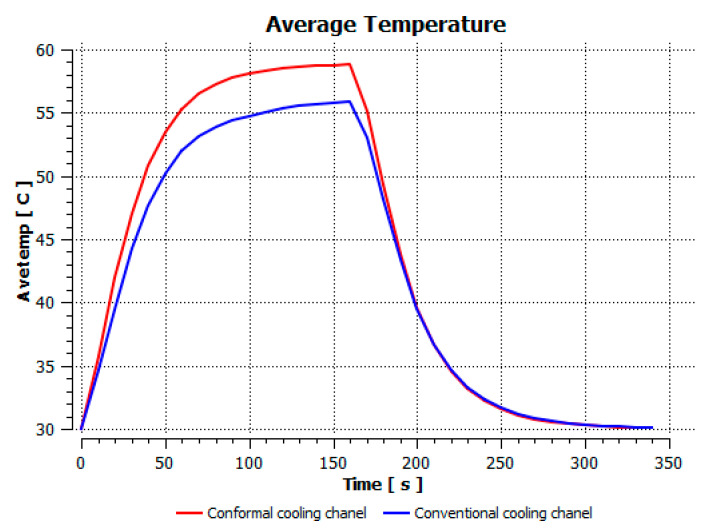
Average temperature.

Figure 11 reports the average temperature of the mold during the heating process. This temperature history was collected during the first cycles with the initial mold temperature at 30 °C to compare the different heating and cooling effects of the traditional and conformal cooling channels. To compare the conformal cooling channel with the traditional case, the average temperatures on the cavity surfaces (Figure 7) were collected. By using the CFX module in the ANSYS software, the average temperature was calculated by the function:

ave(*Temperature*)@Gettemp − 273.15[K]

In the formula:

- Ave(): Find the average variable (in this formula Temperature)
- Gettemp: Name of the green surface domain as in Figure 7.

This comparison showed that the temperature for both molds increased rapidly in the first 100 s. However, during the heating process, the temperature of the mold using a conformal cooling channel was always higher than the other. Thus, the conformal cooling channel demonstrated better effectiveness than the conventional cooling channel. The average temperature of the mold peaked at 58.78 °C, the maximum temperature on the mold surface was 63.4 °C, and the minimum temperature was 54.66 °C. Meanwhile, with the conventional cooling channel, the average temperature in the steady state was 56.63 °C, and the maximum and minimum temperatures at the measurement surface were 61.74 °C and 53.18 °C, respectively. Regarding temperature distribution, in the mold using a conformal cooling channel, the high-temperature surface (greater than 61 °C) occupied a more extensive area than in the mold using a conventional cooling channel, where the high-temperature area (greater than 61 °C) covered only a small space. This indicated that temperature distribution over the injection mold was improved when using a conformal cooling channel.

Table 12 reveals that the temperature distribution with the conventional cooling channel was more significant than with the conformal cooling channel, from the start of the process up to 18 s, and was almost equal in the two types of cooling channel when the mold temperature reached a steady state. This can be explained by the fact that the maximum temperature when using a conventional cooling channel was lower than that with a conformal cooling channel, and the same was true for the minimum temperature.

Table 13 shows the temperature distribution in each section. The value in the simulation was taken automatically at the cavity surfaces (Figure 7) using the Ansys formula.

Maximum temperature:

maxVal(*Temperature*)@Gettemp

Minimum temperature:

minVal(*Temperature*)@Gettemp

In these formulas:

- maxVal(): Maximum value of variable
- minVal(): Minimum value of variable

These results show that the temperature decreased from where the mold surface was in contact with the hot fluid at the top surface. The temperature reductions in the mold using the conformal cooling channel were always smaller than when using the straight cooling channel; for example, in sections A-A and B-B, the temperature dropped from 72.05 °C to 61.6 °C in the conformal cooling channel mold and 70.9 °C to 58.5 °C in the mold using a straight cooling channel. Thus, the temperature decreases in the two types of molds were 10.45 °C and 12.4 °C, respectively. Sections C-C and D-D had temperature drops in the molds with the conformal cooling channel and straight cooling channel of 10.2 °C and 16.6 °C, respectively. These results prove that a conformal cooling channel is more effective than a straight cooling channel in the heating mold process.

For the cooling step, results show that the average temperature over the two molds in the cooling process was similar, as in Table 14. However, when closely looking at the simulation results, the area with lower temperature was larger on the conformal cooling channel mold than with the conventional mold. It means that the conformal cooling channel mold could support a better cooling area than the traditional cooling channel.

### 2.11. Experiments

In order to verify the simulation results, a model mold cavity was fabricated. For various purposes, the actual model had some differences from the simulation model, but this modification did not significantly affect the final result. Two models were fabricated, one using a conformal cooling channel, and another using a straight cooling channel. The model using a straight cooling channel was implemented using the traditional method (e.g., milling, drilling, grinding). However, the process has different for the model with the conformal cooling channel. The cooling channel in this mold was fabricated separately, as it cannot be easily created by drilling. This cooling channel was formed of two half-channels, joined by welding.

**Figure 12:**
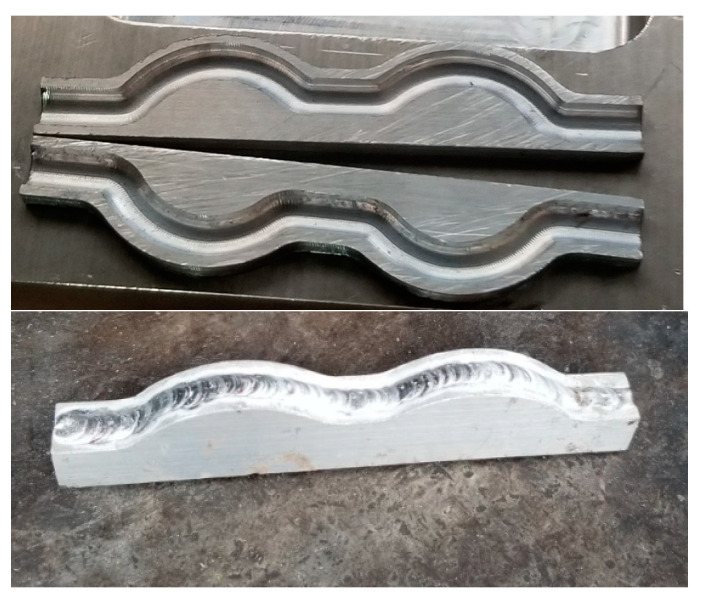
Conformal cooling channel model.

**Figure 13:**
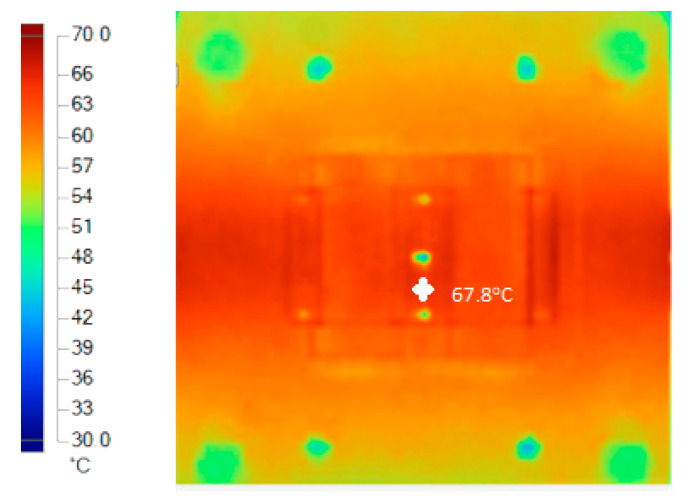
Straight cooling channel mold temperature in the heating process.

**Figure 14:**
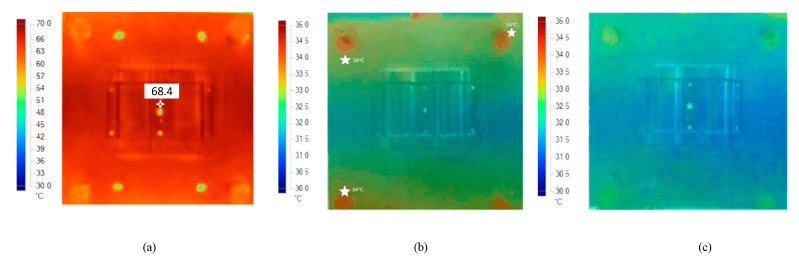
Cavity temperature distribution at the end of heating by (**a**) conformal cooling channel and at the end of cooling with (**b**) traditional cooling channel and (**c**) conformal cooling channel.

**Figure 15:**
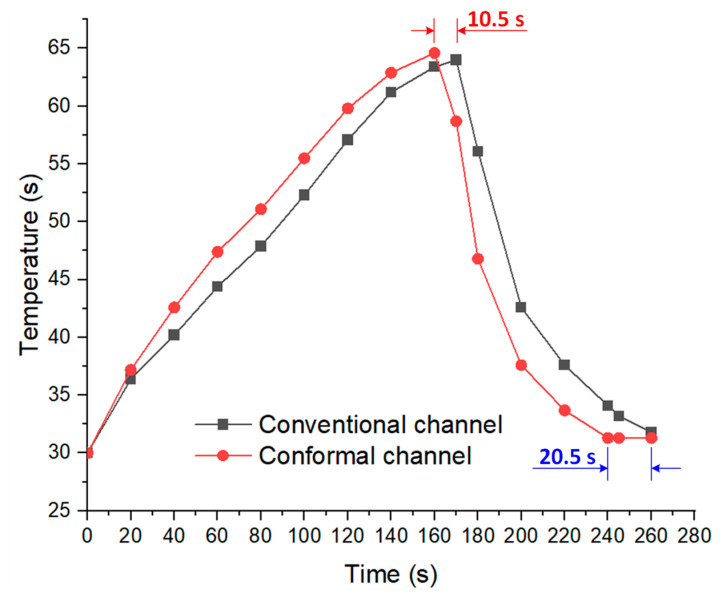
Temperature history throughout the experiment.

**Figure 16:**
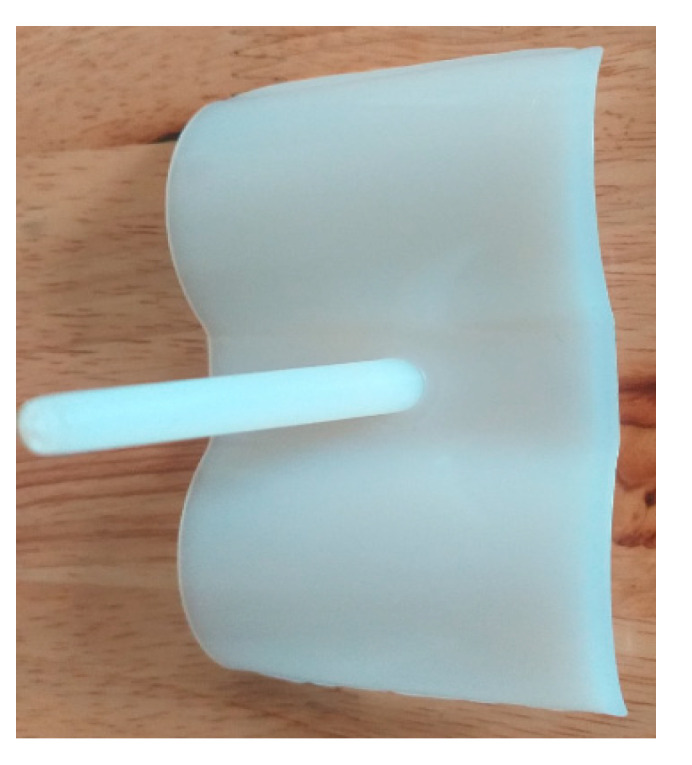
Molded part for testing the molding process with conformal cooling channel.

The experiment was implemented after the molds were finished. The experiment was implemented on both molds, in order to compare the effectiveness of the conformal cooling channel mold. Hot water (80 °C, as in the simulation setup) was pumped into the mold through the temperature control machine and the water supply system. When the temperature at the mold surface reached a steady state, the temperature on the mold surface does not change. Figure 12 show the geometry of 3D cooling channel, which was optimized and will be used for experiment. Figure 13, Figure 14, Figure 15 and Figure 16 depict the experimental results. For observing the temperature distribution of mold surfasce, the infrared camera Fluke TiS60 was applied. The Fluke TiS60 is a high-performance thermal imaging camera designed for professional use in a wide range of applications. It features a high-resolution infrared detector with a 320 × 240-pixel resolution, a temperature measurement range of −20 °C to +1200 °C.

It can be easily recognized, from Figure 13 and Figure 14, that the temperature on the conformal cooling channel was higher than that on the straight cooling channel, and the temperature on the conformal cooling channel mold was distributed more uniformly. The maximum temperature on the conformal cooling channel mold was nearly 68.4 °C (at the top of the wave on the mold, marked by the white point on the Figure 14a), and the average temperature reached 64.6 °C. Meanwhile, the corresponding results for the straight cooling channel mold were 67.8 °C (marked by the white point on Figure 13)and 64 °C, respectively. In order to reach the steady state, the conformal cooling channel mold took 160 s, while the straight cooling channel mold took 180 s to reach this state. This result demonstrates that the CCC is more effective than a straight channel in the mold-heating process.

The simulation results showed that the average temperatures on the mold surface of the CCC and straight cooling channel molds, respectively, were 58.78 °C and 56.63 °C. As the experiment results were 64 °C and 63 °C, the standard deviation was 5.22 °C (8.8%) and 6.37 °C (11.2%), respectively. Comparing the simulation and environmental conditions, this result can be accepted as the setup in the simulation assumed perfect conditions, while in the experiment certain errors may derive from the mold material, which may have different properties. Furthermore, there may have been errors arising from the conformal cooling channel, as it was formed from two half-channels; thus, it may have been slanted due to welding of the two sides. 

Comparing the difference between the effectiveness of two cooling types in the cooling process, the mold using a conformal cooling channel presented the better result. When looking at the experimental results in Figure 14b,c, the results for the straight cooling channel shows that there were some points on the mold surface where the temperature reached nearly 34 °C (marked in white in Figure 14b). However, as shown in Figure 14c, the temperature on the mold surface was lower than the mold that used a straight cooling channel during cooling, and there was no point on the surface of the conformal cooling channel mold where the temperature was greater than 34 °C. The average temperature during the cooling process was approximately 30.5 °C.

Returning to the comparison between the simulation and experimental results, in the simulation, the average temperature on the mold surfaces was approximately 30 °C, and the simulation showed little difference. The average temperature on the conformal cooling channel mold was nearly 30.5 °C, while on the other mold this result was higher (32.5 °C). This deviation was relatively slight, and can be considered negligible.

Figure 15 shows the temperature history for both molds during the experiment. From the start to the end of the heating process, the red line indicates that the temperature on the mold using the conformal cooling channel was higher than that on the other mold. Afterwards, during the cooling step, the black line is above the green line, indicating that the cooling efficiency of the mold using a straight cooling channel was lower than the mold using the conformal cooling channel. 

Product testing involved final inspection of the mold assembly and structure before testing. The necessary plastic, PA 6–30%, was prepared and the SHINE-WELL-120B injection-molding machine was used for the testing process. The mold was mounted onto the machine, the machine’s operation checked, and the plastic material prepared for injection molding. The product was molded to completion using various molding parameters as in Figure 16, including an injection pressure of 30 kg/cm^2^, nozzle temperature of 270 °C, injection speed of 14 mm/s, injection time of 2 s, and cooling time of 8 s. The main dimensions of the injection mold are shown in Table 15.

## 3. Conclusions

In this study, the Taguchi approach and the principal component analysis method were used to determine the optimal shape for the conformal cooling channel in an injection mold. The Taguchi method results indicated that of all the parameters the cooling channel distance and diameter have the most significant effect on heat transfer from the cooling channel to the mold surface. Then, principal component analysis was conducted to decrease the dimensions of the data. In the considered problem, this method was used to normalize two responses into a multi-response performance index, making the optimization problem more accessible. The optimization result showed better effectiveness in terms of temperature distribution over the mold when using the designed conformal cooling channel, compared with a conventional cooling channel. The conformal cooling channel could provide an effective solution for the injection-mold heating process, without adding more components or features to the mold.

## Figures and Tables

**Table 1:** Design parameters for the cooling channel.

Part Thickness w	Channel Diameter d
2 mm	8–10 mm
4 mm	10–12 mm
6 mm	12–15 mm
c = 2–3 d, b = 3–4 d.	

**Table 2:** Conformal cooling channel design parameter ranges.

	Lower Bound	Upper Bound	Unit
H3	35	40	mm
H4	50	55	mm
V5	5	15	mm
V6	15	23	mm
V7	16	24	mm
D1	8	10	mm
FD1	24	40	mm

**Table 3:** Thermal properties for each material.

	Density (kg/m^3^)	Specific Heat (J/kg·K)	Thermal Conductivity (W/m·K)	Heat Transfer Coefficients (W/m^2^·K)
Water	997.0	4181.7	0.6	5200
Aluminum	2720	903	205.00	13

**Table 4:** Total Degrees of Freedom.

Factor	Degrees of Freedom
Overall mean	1
H3, H4, V5, V6, V7, D1, FD1	(3 − 1) × 7 = 14
Interaction	0
Total DOF	1 + 14 = 15

**Table 5:** Design of Experiments.

	Orthogonal Array							Factor						
No	F1	F2	F3	F4	F5	F6	F7	H3	H4	V5	V6	V7	D1	FD1
1	1	1	1	1	1	1	1	35	50	5	15	16	8	24
2	1	1	1	1	2	2	2	35	50	5	15	20	9	32
3	1	1	1	1	3	3	3	35	50	5	15	24	10	40
4	1	2	2	2	1	1	1	35	52.5	10	19	16	8	24
5	1	2	2	2	2	2	2	35	52.5	10	19	20	9	32
6	1	2	2	2	3	3	3	35	52.5	10	19	24	10	40
7	1	3	3	3	1	1	1	35	55	15	23	16	8	24
8	1	3	3	3	2	2	2	35	55	15	23	20	9	32
9	1	3	3	3	3	3	3	35	55	15	23	24	10	40
10	2	1	2	3	1	2	3	37.5	50	10	23	16	9	40
11	2	1	2	3	2	3	1	37.5	50	10	23	20	10	24
12	2	1	2	3	3	1	2	37.5	50	10	23	24	8	32
13	2	2	3	1	1	2	3	37.5	52.5	15	15	16	9	40
14	2	2	3	1	2	3	1	37.5	52.5	15	15	20	10	24
15	2	2	3	1	3	1	2	37.5	52.5	15	15	24	8	32
16	2	3	1	2	1	2	3	37.5	55	5	19	16	9	40
17	2	3	1	2	2	3	1	37.5	55	5	19	20	10	24
18	2	3	1	2	3	1	2	37.5	55	5	19	24	8	32
19	3	1	3	2	1	3	2	40	50	15	19	16	10	32
20	3	1	3	2	2	1	3	40	50	15	19	20	8	40
21	3	1	3	2	3	2	1	40	50	15	19	24	9	24
22	3	2	1	3	1	3	2	40	52.5	5	23	16	10	32
23	3	2	1	3	2	1	3	40	52.5	5	23	20	8	40
24	3	2	1	3	3	2	1	40	52.5	5	23	24	9	24
25	3	3	2	1	1	3	2	40	55	10	15	16	10	32
26	3	3	2	1	2	1	3	40	55	10	15	20	8	40
27	3	3	2	1	3	2	1	40	55	10	15	24	9	24

**Table 6:** Simulation results and S/N ratio for each response.

	Result		S/N Ratio	
No	P8	P9	S/N-P8	S/N-P9
1	25.35	61.41	−28.078	35.7643
2	20.84	65.37	−26.376	36.3075
3	16.20	68.84	−24.188	36.7568
4	21.16	59.11	−26.51	35.4335
5	16.43	62.28	−24.312	35.8874
6	12.46	65.16	−21.909	36.2801
7	20.08	56.78	−26.056	35.0832
8	14.28	59.41	−23.094	35.4769
9	9.78	61.35	−19.805	35.7556
10	14.62	63.79	−23.3	36.095
11	23.11	62.10	−27.275	35.8616
12	16.04	60.78	−24.104	35.6756
13	10.70	61.77	−20.59	35.8158
14	20.70	59.42	−26.32	35.4789
15	15.16	56.81	−23.616	35.0879
16	16.21	67.18	−24.194	36.5448
17	26.28	64.37	−28.392	36.1738
18	19.39	63.55	−25.753	36.0621
19	16.33	61.73	−24.26	35.8094
20	8.76	58.71	−18.846	35.3738
21	18.07	57.24	−25.14	35.1542
22	21.80	67.00	−26.768	36.5219
23	15.00	65.58	−23.524	36.3356
24	25.73	63.37	−28.208	36.0377
25	16.78	63.74	−24.496	36.0884
26	11.39	61.31	−21.13	35.7501
27	22.48	59.50	−27.036	35.4904

**Table 7:** The explained variation and eigenvalues of principal components.

Eigen Analysis of the Correlation Matrix		
	$\vartheta_{1}$	$\vartheta_{2}$
Eigenvalue	1.0928	0.9072
Proportion	0.546	0.454
Cumulative	0.546	1.000

**Table 8:** MPI values for responses.

No	MPI
1	0.06419
2	0.16775
3	0.31122
4	0.19392
5	0.33787
6	0.49954
7	0.24194
8	0.445
9	0.67693
10	0.40424
11	0.11967
12	0.36205
13	0.61631
14	0.20615
15	0.42238
16	0.31952
17	0.02405
18	0.22404
19	0.34498
20	0.76369
21	0.30683
22	0.12992
23	0.3777
24	0.04329
25	0.31597
26	0.57908
27	0.15263

**Table 9:** Mean MPI for each level.

	Levels		
	1	2	3
H3	0.3245	0.2978	0.3329
H4	0.3141	0.3122	0.3289
V5	0.1836	0.3274	0.4442
V6	0.313	0.3329	0.3092
V7	0.2904	0.3336	0.3311
D1	0.3562	0.3084	0.2906
FD1	0.1476	0.3036	0.504

**Table 10:** Optimum levels and values for each parameter.

Optimum		
Factors	Levels	Value
H3	3	40
F4	3	55
V5	3	15
V6	2	19
V7	2	20
D1	1	8
FD1	3	40

**Table 11:** ANOVA for MPI means.

Source	DF	Seq SS	Adj SS	Adj MS	F	P
H3	2	0.00602	0.00602	0.00301	1.29	0.311
H4	2	0.00152	0.00152	0.00076	0.32	0.729
V5	2	0.30679	0.30679	0.1534	65.6	0
V6	2	0.00292	0.00292	0.00146	0.62	0.552
V7	2	0.01059	0.01059	0.00529	2.26	0.147
D1	2	0.02074	0.02074	0.01037	4.44	0.036
FD1	2	0.57449	0.57449	0.28725	122.84	0.000
Residual Error	12	0.02806	0.02806	0.00234		
Total	26	0.95113				
S	R-Sq	R-Sq (adj)				
−0.0467	97.15%	93.82%				

**Table 12:** Temperature distribution on mold surface during the heating process.

Heating Time	Conformal Cooling Channel	Conventional Cooling Channel
10 s	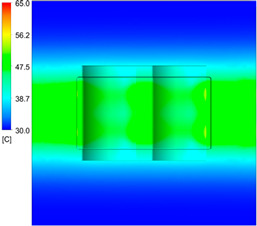	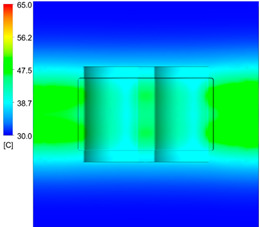
50 s	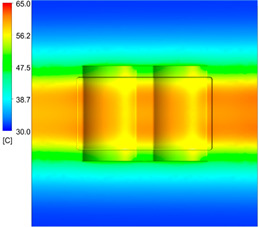	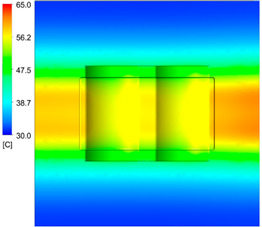
100 s	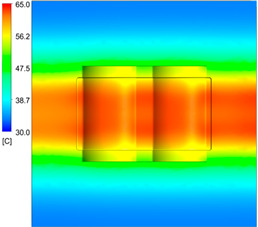	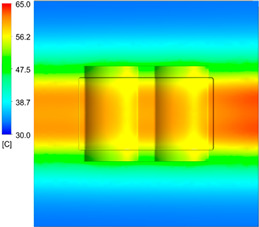
160 s	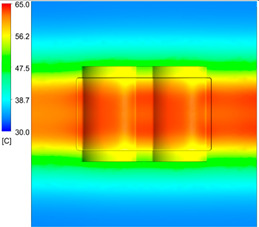	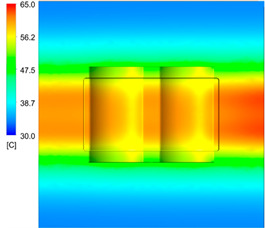
Steady state	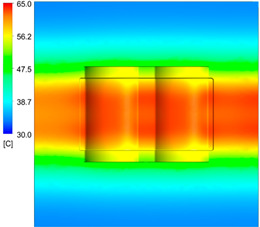	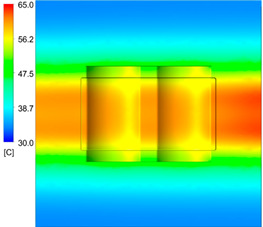

**Table 13:** Temperature distribution in each section.

	Conformal Cooling Channel	Conventional Cooling Channel
	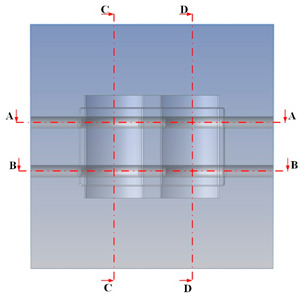	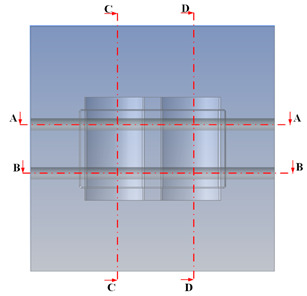
A-A	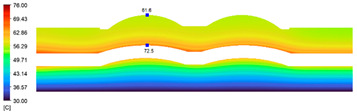	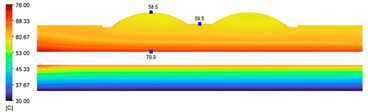
B-B	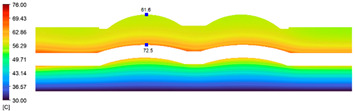	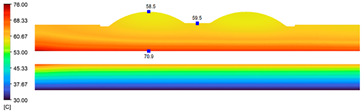
C-C	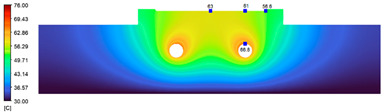	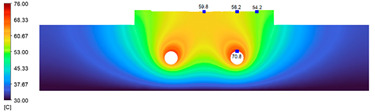
D-D	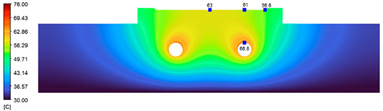	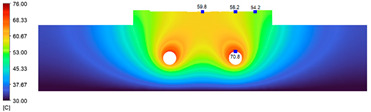

**Table 14:** Temperature distribution on mold surface during the cooling process.

Conformal Cooling Channel	Conventional Cooling Channel
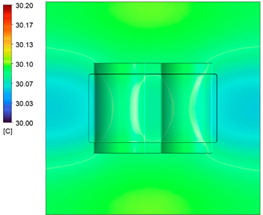	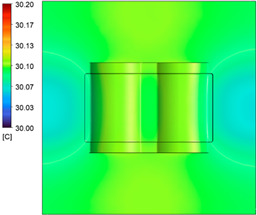

**Table 15:** The main parts dimension of injection mold.

No.	Mold Elements	Element Size	Quantity
1	Adjustable collar	Φ100 × 10	1
2	Top clamp plate	250 × 200 × 20	1
3	Cavity plate	200 × 200 × 30	1
4	Core plate	200 × 200 × 40	1
5	Support cushion	200 × 60 × 30	2
6	Retainer plate	200 × 120 × 13	1
7	Ejector plate	200 × 120 × 15	1
8	Bottom clamp plate	250 × 200 × 20	1
9	3D cooling channel plate	120 × 23 × 6	4

## Data Availability

The data used to support the findings of this study are available from the corresponding author upon request.
